# Intramedullary Spinal Cord Abscess With Brain Abscess Secondary to Empyema: A Report of a Rare Case

**DOI:** 10.7759/cureus.104151

**Published:** 2026-02-23

**Authors:** Miyu Amano, Tatsunori Kiriu, Satoshi Mukaida, Misato Saika, Yoshikazu Kotani

**Affiliations:** 1 Department of Respiratory Medicine, Hyogo Prefectural Awaji Medical Center, Sumoto, JPN

**Keywords:** anaerobic bacterial infection, antimicrobial therapy, brain abscess, empyema, intramedullary spinal cord abscess, streptococcus constellatus

## Abstract

Intramedullary spinal cord abscess (ISCA) is a rare condition that requires prompt intervention. We report the case of a 60-year-old Japanese man with type 2 diabetes who was diagnosed with ISCA and a concomitant brain abscess during antibiotic treatment for empyema. Antimicrobial therapy alone led to remission and obviated the need for surgery following diagnosis. At the one-year follow-up, the patient showed no sequelae or recurrence. Early diagnosis and appropriate antimicrobial therapy are crucial for improving prognosis and minimizing permanent neurological deficits.

## Introduction

Intramedullary spinal cord abscess (ISCA), a rare central nervous system condition, was first reported in 1830. From 1830 to 1944, its mortality rate was 90%, which significantly decreased to 4% between 1998 and 2007 [1], due to advances in antimicrobial therapy.

Despite these advances, a standardized protocol for antimicrobial therapy remains elusive, leading to variability in drug selection, dosage, and duration across cases. Additionally, while some reports suggest that antimicrobial therapy alone can be effective, the optimal treatment strategy, medical versus surgical, remains debated, particularly for cases involving a concurrent brain abscess.

Infections can reach the spinal cord via three main pathways [2]. The first is hematogenous spread from a distant site of infection, such as endocarditis, pneumonia, or urinary tract infection, which leads to bacteremia and subsequent seeding of the spinal cord. The hematogenous route is considered the primary route in adults. The second is a direct extension from an adjacent focus of infection. The third is direct implantation, such as from trauma or a procedure.

This case report details a case of empyema-associated ISCA with a concomitant brain abscess, successfully managed with antimicrobial therapy. To our knowledge, this is the first documented case of ISCA originating from empyema.

## Case presentation

A 60-year-old Japanese man presented to the hospital with a 3-month history of right back pain as his chief complaint. His medical history included hypertension, type 2 diabetes mellitus, and dyslipidemia. Chest computed tomography (CT) revealed right pleural effusion and right lower lobe atelectasis due to compression. Following a diagnosis of bacterial pleurisy, oral antibiotic therapy was initiated; however, it proved ineffective. The patient was subsequently admitted with fever, neck pain, motor and sensory disturbances in the right upper limb, and urinary retention.

Upon admission, breath sounds were diminished in the right lung. The right upper extremity exhibited sensory disturbances, primarily in the C5-C6 dermatomes. The Manual Muscle Testing (MMT) score was 1. Furthermore, the patient reported spontaneous neck pain upon retroflexion, and the Jolt Accentuation test was negative. Laboratory findings revealed leukocytosis (white blood cell (WBC) count of 11,960/µL) and elevated C-reactive protein (CRP) levels (5.36 mg/dL).

**Figure 1:**
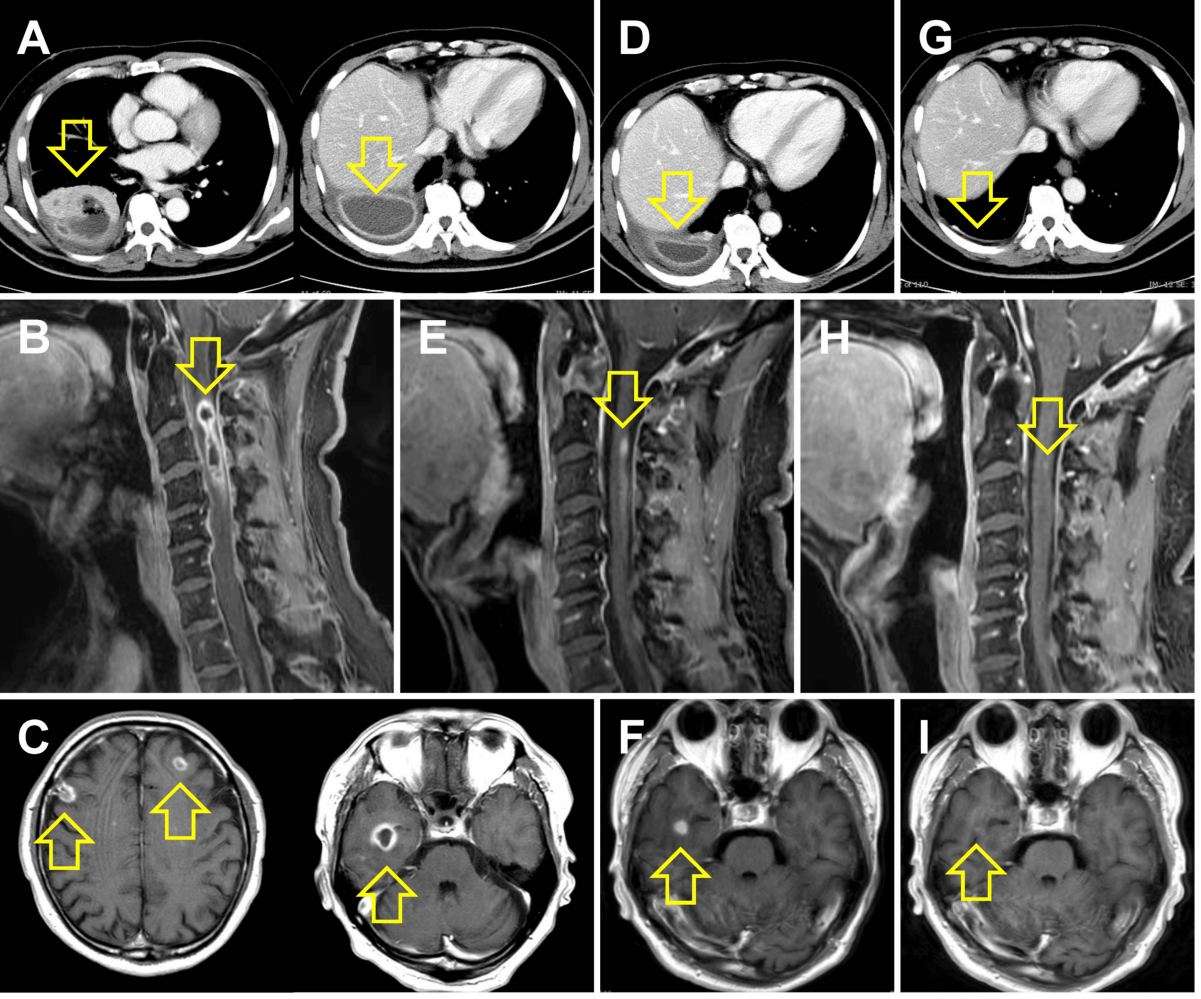
Imaging findings (A) Initial chest computed tomography (CT) scans obtained at admission showing an indistinct demarcation between the right lower lobe and right pleural effusion, with air presence within the effusion, and a segmental pleural effusion characterized by thick septations. (B) Spinal sagittal contrast-enhanced T1-weighted (CE-T1WI) magnetic resonance imaging (MRI) obtained at admission reveals an occupying lesion with peripheral contrast enhancement in the C2-4 region. (C) Brain axial CE-T1WI MRI indicates ring-enhancing lesions located in both the frontal lobes and the right temporal lobe. (D, G) Subsequent CT demonstrated a significant reduction in the size of the latter lesion at (D) 1 month and (G) 4 months following treatment initiation. (E, F, H, I) Subsequent MRI indicated a marked decrease in the size of these lesions at 1 month (E, F) and 4 months (H, I) following treatment initiation.

Chest radiography showed decreased translucency in the right lower lung field compared to the initial examination. Chest CT revealed a loculated pleural effusion with thick septa, indicating disease progression (Figure 1A). Pleural fluid analysis revealed an elevated cell count (1381458/µL) with a neutrophil predominance (78.5%). *Streptococcus constellatus* (*S. constellatus*) was isolated from the pleural fluid cultures. Antibiotic susceptibility testing indicated susceptibility to penicillins, cephalosporins, quinolones, tetracyclines, carbapenems, and vancomycin, but resistance to macrolides and lincomycin (Table 1). No pathogens were detected in blood or cerebrospinal fluid cultures. Spinal cord magnetic resonance imaging (MRI) revealed an occupying lesion at the C2-4 region with peripheral contrast enhancement (Figure 1B). Furthermore, brain MRI showed similar ring-enhancing lesions in the temporal and frontal lobes (Figure 1C). Consequently, the patient was diagnosed with ISCA and brain abscesses secondary to empyema.

**Table 1:** Drug susceptibility to Streptococcus constellatus MIC, minimum inhibitory concentration

Antibiotic	Pleural effusion culture	
	Susceptibility	MIC (µg/mL)
Penicillin G	Susceptible	0.06
Ampicillin	Susceptible	≤ 0.06
Ceftriaxone	Susceptible	0.25
Cefepime	Susceptible	≤ 0.5
Levofloxacin	Susceptible	0.5
Meropenem	Susceptible	≤ 0.12
Vancomycin	Susceptible	1
Minocycline	Susceptible	2
Chloramphenicol	Susceptible	≤ 4
Erythromycin	Resistant	> 2
Azithromycin	Resistant	> 4
Clindamycin	Resistant	> 1

During outpatient treatment, the patient received levofloxacin (500 mg daily) for 1 week, galenoxacin (400 mg daily) for 4 weeks, and a combination of cephalexin (100 mg daily) and clindamycin (600 mg daily) for 8 weeks. Upon hospital admission, the regimen was changed to ampicillin/sulbactam (ABPC/SBT) at 3 g every 6 hours.

Following consultations with the chest surgery, orthopedic surgery, and neurology departments regarding empyema, ISCA, and brain abscess, respectively, a decision was made to continue antimicrobial therapy. On day 4, the regimen was modified to better target the brain abscesses. The dose was adjusted to 2 g of ABPC every 4 hours, delivered as 2 g ABPC every 8 hours in conjunction with the existing ABPC/SBT infusions, achieving a total daily dose of 12 g ABPC. On day 26, the patient developed a fever of > 39 °C with chills. Although the WBC count was normal, the CRP level was significantly elevated at 13.33 mg/dL. Chest CT showed a reduction in empyema size, suggesting ABPC/SBT was effective (Figure 1D). Consequently, antimicrobial therapy was changed to meropenem (2 g every 8 hours). The fever resolved rapidly, and the inflammatory response improved. On day 33, MRI revealed a reduction in the size of both the ISCA and brain abscess (Figures 1E, 1F). Considering the documented efficacy of the prior regimen, the therapy was switched back to ABPC/SBT. However, on day 36, the patient developed a fever of > 38 ℃, consistent with a drug fever attributed to ABPC/SBT.

**Figure 2:**
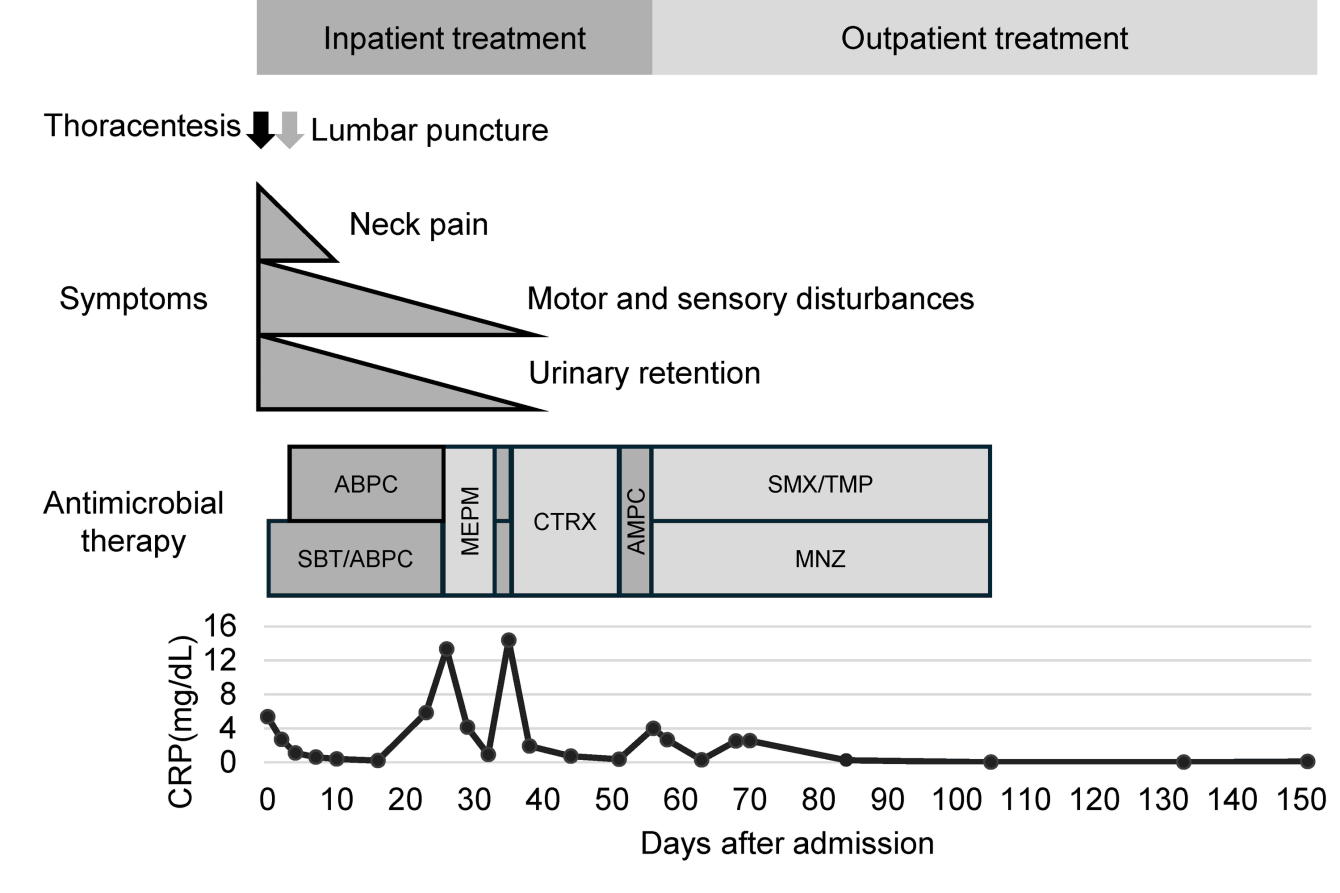
Treatment and clinical course Top panel: procedures and clinical symptoms. Neck pain, motor and sensory disturbances, and urinary retention improved following antimicrobial treatment. Middle panel, schedules of antimicrobial treatment. Bottom panel: laboratory findings. ABPC/SBT: ampicillin/sulbactam, MEPM: meropenem, CTRX: ceftriaxone, AMPC: amoxicillin, SMX/TMP: sulfamethoxazole/trimethoprim, MNZ: metronidazole, CRP: C-reactive protein

Consequently, the regimen was changed to ceftriaxone (CTRX; 2 g every 12 hours). On days 42 and 49, the erythrocyte sedimentation rate (ESR) was normalized, prompting the cessation of intravenous therapy and a transition to oral ampicillin (2000 mg daily). However, on day 57, the CRP level rose to 4.00 mg/dL in the absence of fever. This elevation was suspected to be a reaction to the penicillin-based antibiotic. Therefore, antimicrobial therapy was modified to a combination of sulfamethoxazole/trimethoprim (3600/720 mg) daily and metronidazole (MNZ; 1500 mg daily). This change led to the resolution of the inflammatory response, and the patient was discharged on day 59. Antimicrobial therapy was completed on day 106, with no subsequent neurological sequelae. This decision was based on normalized ESR and a significant improvement in imaging (Figures 1G-1I). The clinical course is summarized in Figure 2.

## Discussion

To our knowledge, this is the first documented case of ISCA and brain abscess originating from empyema. Although the condition persisted for over a month from initial presentation, remission was achieved with antimicrobial therapy alone, without surgical intervention. No underlying immunocompromising conditions, other than type 2 diabetes, were identified. The causative organism was *S. constellatus*.

*S. constellatus* belongs to the *Streptococcus anginosus* group (SAG), which typically colonizes the upper respiratory, digestive, and reproductive tracts [3]. A comparison of SAG species indicates that *S. constellatus* is more strongly associated with bacteremia than with abscess or empyema formation [4]. Given that *S. constellatus* in empyema is often accompanied by anaerobic bacteria, selecting antimicrobials against both is crucial [5]. In this case, we hypothesized that the initial treatment was ineffective against anaerobic bacteria, leading to the progression of empyema and bacteremia to the ISCA and brain abscess. Notably, because no viable specimens from the ISCA and brain abscess were available for culture, this pathogenesis remains speculative. The initial treatment with ABPC/SBT was chosen based on the identification of *S. constellatus*. An alternative empirical regimen could have been CTRX plus MNZ [6].

ISCA is classified by time since symptom onset into acute, subacute, and chronic phases. However, symptoms such as fever, back pain, and spinal nerve disorders may not be present in all phases [7]. Secondary ISCA typically arises from factors such as dermal sinus infections, sepsis, contiguous spread from adjacent infection, and cryptogenic sources. Prompt diagnosis and treatment are essential, as most patients suffer persistent neurological deficits [8]. However, documented evidence of cases are available, where improvement has been achieved with antimicrobial therapy alone, obviating the need for surgery [1,9].

However, when symptoms deteriorate rapidly, early surgical intervention improves neurological prognosis [8,10]. Therefore, early surgery can be beneficial, and determining its optimal timing is critical.

In this case, a key concern was that if antimicrobial therapy failed, rapid ISCA progression could compromise the upper cervical spinal cord and lead to respiratory dysfunction. Consequently, multidisciplinary consultations were held to evaluate the need for chest drainage or video-assisted thoracoscopic surgery for empyema, as well as potential surgical excision of the ISCA. The presence of neck pain upon extension also raises concerns about the feasibility of performing surgery under general anesthesia. Given the uncertainty risk of creating a bronchopleural fistula, chest drainage was withheld. Because the *S. constellatus* isolated from the pleural fluid showed a favorable susceptibility profile, a trial of antimicrobial therapy was initiated.

Treatment was completed without neurological sequelae. Although surgical drainage combined with long-term antimicrobial therapy is standard for abscesses, remission can be achieved with antimicrobial therapy alone, as demonstrated in our case. Therefore, a systematic evaluation of risks and benefits is imperative for each case to select the optimal treatment and facilitate timely surgical decisions following diagnosis.

For central nervous system infections, careful selection of antimicrobial agents and routes of administration is essential. While the optimal duration of therapy is debatable, a minimum of six weeks of intravenous therapy is generally recommended [2,11]. Follow-up for at least one year is advised, given a reported occurrence rate of 25% [2]. In our patient, a normalized ESR served as an adjunctive criterion for switching to oral therapy, which was continued until imaging demonstrated lesion stabilization.

One year after treatment initiation, the patient remains free of sequelae or recurrence.

## Conclusions

We encountered a case of spinal and brain abscesses that progressed due to an inadequate initial response to empyema treatment. This instructive case emphasizes the importance of early chest drainage and should be considered in the presence of an anaerobic bacterial infection.
